# Answering questions posed by health system stakeholders using linked administrative health data at the Institute for Clinical Evaluative Sciences (ICES)

**DOI:** 10.23889/ijpds.v1i1.325

**Published:** 2017-04-19

**Authors:** Erika A. Yates, Marian J. Vermeulen, Refik Saskin, J. Charles Victor, P. Alison Paprica, Michael J. Schull

**Affiliations:** 1 Institute for Clinical Evaluative Sciences

## Objectives

There is a growing need to broaden access to administrative health data in order to support decision making and planning by health system stakeholders. An initiative funded by the Ontario Ministry of Health and Long-Term Care, the Applied Health Research Question (AHRQ) portfolio leverages the linked administrative health data holdings and the scientific and clinical expertise at ICES to answer questions generated by stakeholders that will have a direct impact on health care policy, planning or practice.

## Approach

Eligible requesters include government ministries, health care providers and planners. Requests detail the purpose of the research question, the related scientific literature, and the planned use and intended impact of the research findings. An internal review team meets monthly to adjudicate; requests demonstrably needing research findings rapidly are adjudicated on an ad hoc basis. Eligible requests are those that aim to inform evidence-based decision making, do not advocate for a particular answer and are feasible in terms of data availability. All projects are reviewed by the internal privacy office to ensure that use of the administrative health data is in accordance with both data sharing agreements and legislation governing use of personal health information. At no cost to the requesting organization, ICES scientists and research staff formulate the analysis plan, conduct the analysis and prepare the research product (data tables, a slide deck and/or a written report); and, may opt to publish noteworthy findings. All research products must be cleared for risk of re-identification prior to being shared externally.

## Results

Requests have steadily increased from 43 submissions in fiscal year 2012/13, to 59 in 2014/15 and 74 to date in 2015/16. In fiscal year 2014/15, provincial government and government agencies were the most frequent requesters (39%), followed by hospitals and other health care providers (19%), disease advocacy groups (12%) and professional associations (10%). Requests include assessment of health care utilization; health system performance and evaluation; and chronic disease prevalence and treatment. Time to complete reports varies from 5 days to 24 months, depending on project complexity and requirements. Requesters report that AHRQ research findings have influenced decision-making, policy development and health care practice; and have inspired future research.

## Conclusions

This initiative demonstrates the value and feasibility of using the linked administrative health data to answer questions to meet the unique needs of health planners and policymakers, and presents an opportunity for collaboration beyond the academic research community.

